# Chia seed supplementation and inflammatory biomarkers: a systematic review and meta-analysis

**DOI:** 10.1017/jns.2024.70

**Published:** 2024-12-11

**Authors:** Pedram Pam, Sanaz Asemani, Mohammad Hesam Azizi, Parmida Jamilian

**Affiliations:** 1 Student Research Committee, Tabriz University of Medical Sciences, Tabriz, Iran; 2 Faculty of Nutrition and Food Sciences, Tabriz University of Medical Sciences, Tabriz, Iran; 3 Department of Nutrition, School of Public Health, Iran University of Medical Sciences, Tehran, Iran; 4 School of Pharmacy and Bioengineering, Keele University, Staffordshire, UK

**Keywords:** C-reactive protein, Chia seed, Inflammation, Interleukin-6, Tumour necrosis factor-alpha

## Abstract

Chia seeds have gained attention for their potential anti-inflammatory properties, which may be attributed to their high content of omega-3 fatty acids, dietary fibre, and antioxidants. This study aims to provide an overview of the current understanding regarding the effects of chia seeds on inflammatory markers, specifically C-reactive protein (CRP), interleukin-6 (IL-6), and tumour necrosis factor-alpha (TNF-α). A comprehensive literature search was conducted on PubMed, Scopus, Web of Science, Cochrane, and Google Scholar up to June 2024. Randomized controlled trials (RCTs) assessing the effect of chia seed on CRP or/and IL-6 or/and TNF-α. Data were extracted and analysed using a random-effects model, and reported as weighted mean differences (WMD) with 95% confidence intervals (CI). Subgroup and sensitivity analyses were also performed. Four RCTs involving 210 participants were included in the meta-analysis. The results showed that chia consumption significantly decreased CRP (WMD: –0.64 mg/dl; 95% CI: –1.24, –0.04; P = 0.03). But it had no significant effect on IL-6 (WMD: 0.29 pg/dl; 95% CI: –0.40, 0.98; P = 0.41), and TNF-α (WMD: 0.05%; 95% CI: –0.21 to 0.30; P = 0.72). Chia consumption can significantly decrease CRP, but no significant effect was observed on IL-6 and TNF-α. To prove our findings, more studies with a larger sample size are needed.

## Graphical abstract

**Figure:**
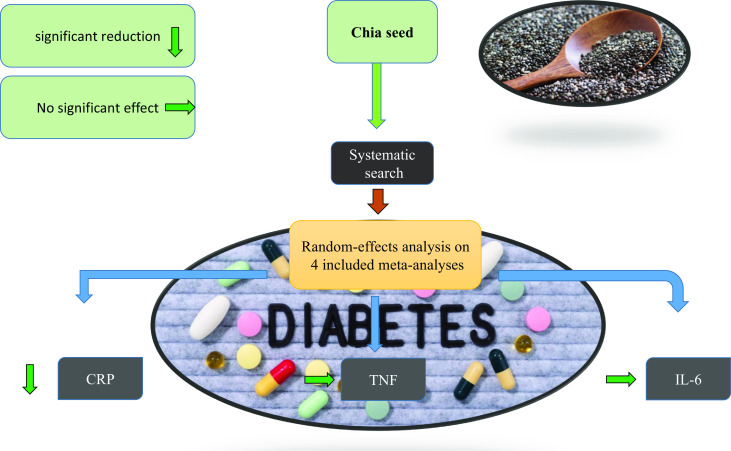

## Introduction

Cellular damage prompts alarm signals in our biological system, triggering an inflammatory response aimed at countering the damage and maintaining internal stability.^(1)^ In conditions like cancer, sepsis, and autoimmune disorders, there is disruption in the regulation of inflammatory processes.^(2)^ Inflammation plays a crucial role after infections or physical trauma by orchestrating tissue repair, restoring equilibrium, and bolstering the host’s defences against external pathogens.^(3)^ The inflammatory cascade begins with a rapid induction phase, followed by pro-inflammatory stages and resolution phases. Disruption in these coordinated phases leads to uncontrolled inflammation, contributing to various inflammatory diseases such as neurological disorders, autoimmune conditions, cancer, sepsis, cardiovascular diseases, and obesity.^(4)^

Chia (Salvia hispanica L.), originally from northern Guatemala and southern Mexico, is an annual herbaceous plant that has spread worldwide.^(5)^ Chia seeds are valued for their functional and nutritional properties, including their ability to form a gel-like consistency when mixed with water due to their high mucilage content. This property enhances the texture of various recipes. Chia seeds are versatile and are used in different forms such as oils, flours, and whole seeds. They are integrated into a wide range of foods such as fruit smoothies, salads, dairy beverages, cereal bars, breads, cookies, yogurts, fruits, and cakes. Furthermore, chia seeds are utilized as effective thickening agents in sauces and soups, making them highly versatile in culinary applications.^(6–8)^

It is noteworthy that chia seeds contain approximately 20% protein.^(9)^ The proteins obtained from digested chia seeds have anti-inflammatory properties. Specifically, these proteins inhibit PPARγ, which in turn reduces the expression of nuclear factor-kappa B (NF-κB), thereby mitigating inflammation.^(10)^ Research indicates that during adipogenesis, digested proteins like albumin and glutelin inhibit the expression of sterol regulatory element-binding protein 1 (SREBP), which is known to activate PPARγ.^(11)^ This reduction in SREBP expression leads to a diminished stimulation of NF-κB, resulting in decreased inflammation.^(10)^ Studies investigating the neuroprotective effects of chia peptides have identified three fractions (1, 1–3, and 3–5 k Dalton) derived from enzymatic hydrolysis of chia proteins. These peptides demonstrate protective and anti-inflammatory effects on nervous system cells, particularly HMC3 microglia cells, by reducing reactive oxygen species (ROS) and inflammatory mediators such as TNF-α, IL-6, H2O2, and NO.^(12)^

Three meta-analyses have been conducted, and published in 2018^(13)^ and 2023.^(14,15)^ The studies by NikPayam *et al.* in 2023 and Teoh *et al.* in 2018,^(13)^ included research that integrated additional supplements alongside chia as interventions, potentially confounding the true impact of chia. Given this limitation, our study specifically focuses on investigations solely examining chia as the intervention. Furthermore, Sarmiento *et al.* ^(15)^ conducted a meta-analysis showing reduced serum CRP levels with chia seed consumption, but they did not evaluate TNF and interleukin as inflammatory indicators. Their analysis included data from 3 publications (5 studies) up to 2021. In contrast, another study combined data from 4 publications (6 studies) and employed a random-effects model to better handle the heterogeneous data observed across the studies, which was deemed more suitable than the fixed model used by Sarmiento *et al.*

Our study stands out for its exclusive focus on chia seeds, unlike previous meta-analyses that included studies with additional supplements.^(13,14)^ In addition, we have compiled a larger data set than previous meta-analyses.^(15)^ Using a random-effects model addresses the heterogeneity among studies, thereby strengthening the reliability of our findings. This meticulous approach aims to advance the understanding of chia’s therapeutic potential in managing inflammatory biomarkers.

## Materials and methods

The current study adhered to the guidelines outlined in the Preferred Reporting Items for Systematic Reviews and Meta-Analyses (PRISMA) statement.^(16)^ The study’s protocol has been officially recorded on PROSPERO with the registration code **CRD42023442343**.

### Search strategy

We conducted a comprehensive literature search on PubMed, Scopus, Web of Science, Cochrane, and Google Scholar up to June 2024. The merge of MESH and non-MESH terms were used as follows: (“chia” OR “chia seed” OR “Salvia hispanica”) AND (“randomized controlled trial”). The details of the search strategy are provided in Supplementary Table 1. To ensure the comprehensive inclusion of relevant studies, we also manually searched the reference lists of eligible studies, as well as relevant reviews and meta-analyses (hand-search method). The language was limited to English studies only.

### Eligibility criteria

The guidelines for selecting studies and conducting the meta-analysis were established using the PICO approach, which involves outlining the Population (P), Intervention (I), Comparison (C), and Outcome (O) criteria for the search process. This meta-analysis incorporated studies meeting the following criteria: (a) randomized controlled trials (RCTs), (b) involving participants aged 18 years and above, (c) reporting at least one of the following outcomes: CRP and/or IL-6 and/or TNF-α.

Studies were excluded if they met any of the following criteria: (a) non-randomized study design, (b) assessed the effects of chia in combination with other interventions, (c) lacked sufficient information regarding the outcomes of interest, (d) had a follow-up period of less than one week, or (e) were conducted exclusively on children, pregnant or lactating women. We used only published studies and did not use grey literature.

### Data extraction

The study selection process was conducted independently by two researchers (P.P and S.A.), with a chief investigator (P.J.) available to resolve any discrepancies. In cases where data were unavailable, we contacted the corresponding author via email to request the necessary information. The following details were extracted from each included study: the first author’s name, year of publication, study location, duration of the study, participant demographics including gender, mean age, and mean body mass index (BMI), study design, the health status of the study population, sample size in each group, chia seed consumption dosage, as well as measurements of CRP and/or IL-6 and/or TNF-α before and after the intervention.

### Data synthesis

The primary outcome was assessed by calculating the mean and standard deviation (SD) differences between before and after supplementation in CRP, IL-6, and TNF-α in intervention and placebo groups. A random-effects model was employed to estimate the overall effect size using restricted maximum likelihood (REML) method. As the measurement units of outcomes were the same, the data were reported as weighted mean difference (WMD). To convert the standard error of the mean (SEM) into SD, the following formula was used: SD = SEM × √n (where n represents the number of participants in each group). The Cochrane’s Q test (with significance set at P < 0.1) and I^2^ statistic were utilized to assess heterogeneity among studies, with an I^2^ value >50% indicating substantial heterogeneity.^(17)^ Predefined subgroup analyses were conducted based on intervention duration, chia seed dosage, gender, and health status to explore potential sources of heterogeneity. Additionally, a sensitivity analysis was performed to examine the impact of individual studies or groups of studies on the overall results. Because the number of observations for each outcome was less than 8, Begg’s and Egger’s tests and visual inspection of the funnel plot were not performed. Statistical analyses were conducted using STATA software (version 14.0; StatCorp, College Station, TX, USA). A significance level of P < 0.05 was predetermined to indicate statistical significance.

### Quality assessment

Version 2 of the Cochrane risk-of-bias tool (RoB2) was used to assess the quality of the included studies (Table 1).^(18)^ This system consists of five criteria to evaluate the risk of bias, which are as follows: Randomization Process, Deviation from Intended Interventions, Missing Outcome Data, Measurement of the Outcome, and Selection of the Reported Result. After evaluating these domains for each study, an overall judgement of ‘low risk’, ‘some concerns’, or ‘high risk’ is assigned to indicate the level of bias in the study. The assessment of the overall risk of bias was determined based on specific criteria: studies were categorized as having a low risk of bias when all domains were deemed to have a low risk; categorized as having some concerns when at least one domain raised some concerns but none were considered high risk; and categorized as high risk of bias if at least one domain was assessed as high risk or if multiple domains raised some concerns.

**Table 1.** Quality assessment

Studies	Randomization Process	Deviation from Intended Interventions	Selection of the Reported Result	Measurement of the Outcome	Missing Outcome Data	General risk of bias
Nieman *et al.* 2009(A)	U	L	L	L	L	some concerns
Nieman *et al.* 2009(B)	U	L	L	L	L	some concerns
Nieman *et al.* 2012(A)	U	L	L	L	L	some concerns
Nieman *et al.* 2012(B)	U	L	L	L	L	some concerns
Vuksan *et al.* 2017	L	L	L	L	L	Low
Vuksan *et al.* 2007	L	L	L	H	H	High

Abbreviations: U, Unclear risk’ of bias; L, Low risk of bias; H, High risk of bias.

### Certainty assessment

The overall certainty of evidence across the studies was graded according to the guidelines of the GRADE (Grading of Recommendations Assessment, Development, and Evaluation) Working Group. The quality of evidence was classified into four categories, according to the corresponding evaluation criteria: high, moderate, low, and very low.^(19)^

## Results

### Study selection

**Figure 1.**
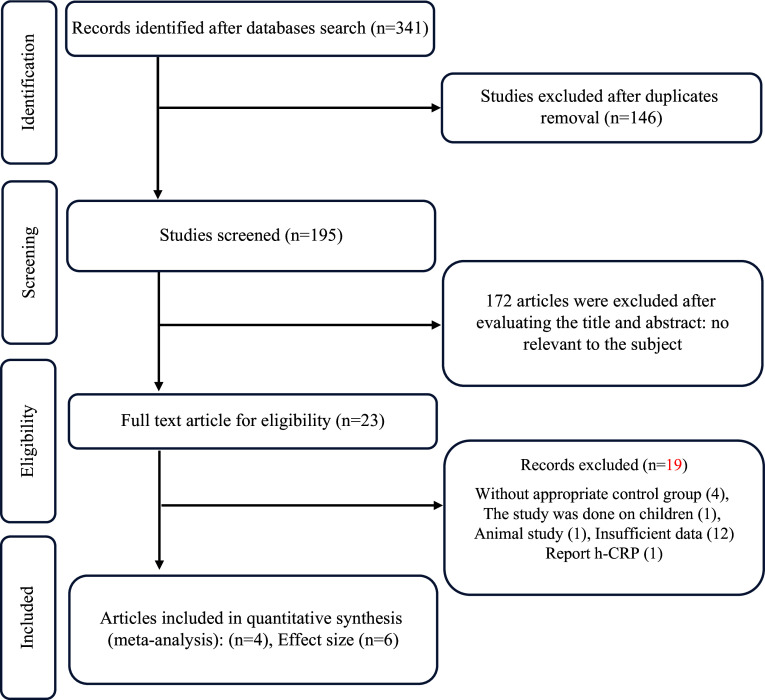
Flow diagram of study selection.

Out of the initial pool of 341 articles identified through the primary search, 146 duplicate studies were removed. Following the screening of titles and abstracts, an additional 195 studies were excluded based on the predetermined inclusion criteria: studies with unrelated titles and abstracts (n = 168), animal studies (n = 21), and review articles (n = 6). As a result, 23 articles remained for full-text screening, and after this evaluation, 19 articles were ineligible for inclusion because they lacked essential information, such as the absence of a control group, focus on paediatric subjects, inclusion of co-supplements, or involvement of animal-based studies. Ultimately, 4 studies (6 effect size) fulfilled all the inclusion criteria and were included in the meta-analysis. The search process and study selection are visually presented in Fig. 1, following the PRISMA flow diagram.

### Study characteristics

Overall, four studies with 6 datasets, comprising 210 participants (106 cases and 104 controls), were included. The included studies were published between 2007 and 2023. The follow-up period ranged from 10^(20)^ to 24^(21)^ weeks, and the sample size of the included studies ranged from 20^(22)^ to 58^(21)^ participants. All of the studies were parallel RCTs, except for one study that had a cross-over design.^(22)^ Selected studies enrolled subjects with type 2 diabetes mellitus^(21,22)^ and overweight.^(20,23)^ The investigations were conducted in the USA^(20,23)^ and Canada.^(21,22)^ One data set enrolled only males, three only enrolled females, and two involved both genders. Characteristics of the included studies are abstracted in Table 2.

**Table 2.** Characteristic of included studies in meta-analysis

studies	Country	Study Design	Participant	Sample size and Sex	Sample size		Trial Duration (Week)	Means Age		Means BMI		chia dose (g/d)
					IG	CG		IG	CG	IG	CG	
Nieman *et al.* 2009(A)	USA	parallel, R, PC, SB	overweight adults	M:28	14	14	12	NR	NR	NR	NR	50
Nieman *et al.* 2009(B)	USA	parallel, R, PC, SB	overweight adults	F:48	25	23	12	NR	NR	NR	NR	50
Nieman *et al.* 2012(A)	USA	parallel, R, PC, DB	overweight women	F:42	16	26	10	60.4 ± 1.6	58.5 ± 1.1	32.9 ± 1.3	33.1 ± 0.9	25
Nieman *et al.* 2012(B)	USA	parallel, R, PC, DB	overweight women	F:40	14	26	10	57.2 ± 1.7	58.5 ± 1.1	33.5 ± 1.5	33.1 ± 0.9	25
Vuksan *et al.* 2017	Canada	parallel, R, PC, DB	overweight and obese patients with type 2 diabetes	M/F (F:40, M:18)	27	31	24	60 ± 2	60 ± 2	31 ± 0.9	30.7 ± 0.7	50
Vuksan *et al.* 2007	Canada	Cross-over, R, PC, SB	T2DM	M/F (F:9, M:11)	10	10	12	64 ± 8	64 ± 8	28 ± 4	28 ± 4	37

Abbreviations: IG, intervention group; CG, control group; DB, double-blinded; SB, single-blinded; PC, placebo-controlled; R, randomized; NR, not reported; F, Female; M, Male; NR, not reported.

### Effect of chia supplementation on CRP

**Figure 2.**
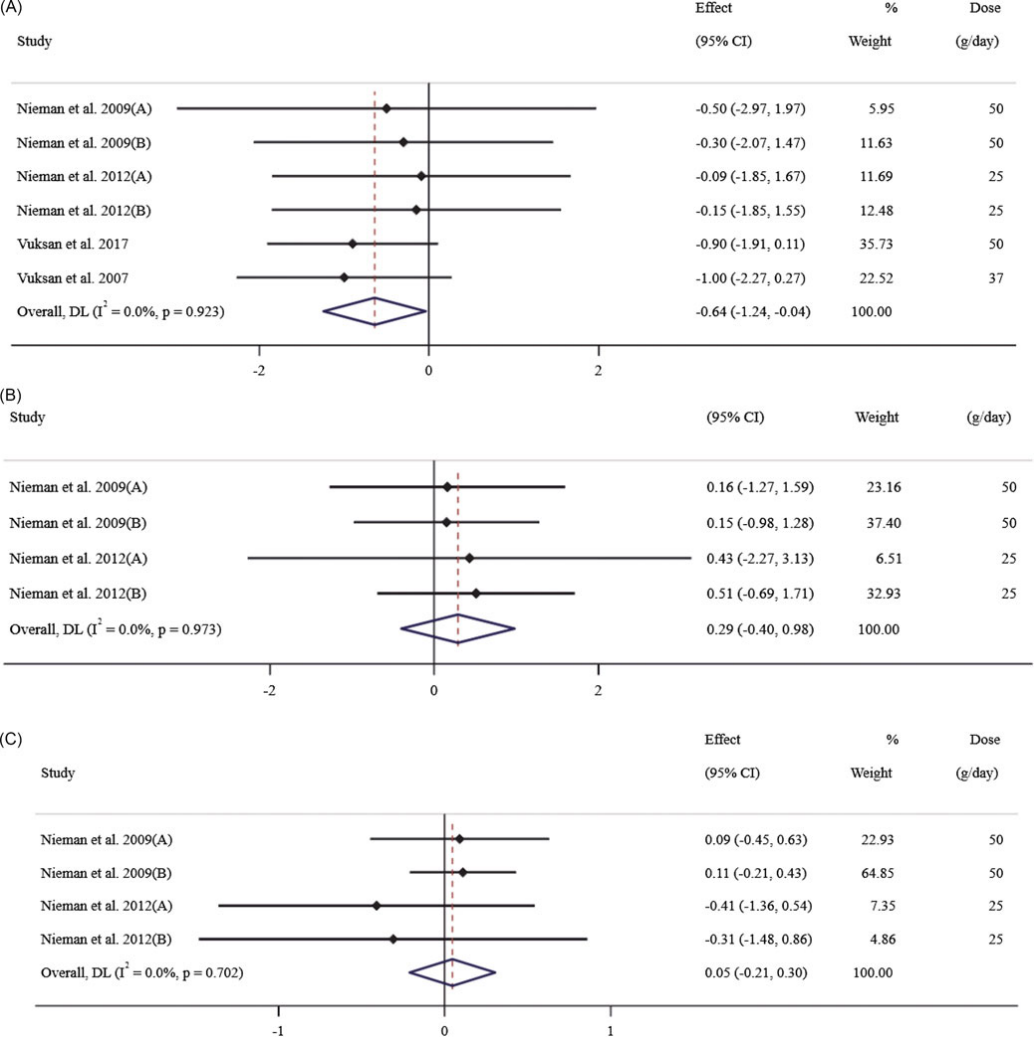
Forest plot for the effects of chia seed ingestion on CRP (A), IL-6 (B), and TNF-α (C).

Four publications (with 6 effect size), containing 210 participants (106 cases and 104 controls), examined the effects of chia on CRP. The overall effect size showed that chia supplementation had a significant effect on CRP reduction (WMD: –0.64 mg/dl; 95% CI: –1.24 to –0.04; P = 0.03) (Fig. 2a), with no degree of heterogeneity (I^2^ = 0%, P = 0.92). The results of the subgroup analysis showed that chia supplementation was effective in decreasing CRP in patients with T2DM, duration of intervention ≥12 weeks, and intervention dosage >35 g/day (Table 3).

**Table 3.** Subgroup analyses of chia on FBS, Insulin, and HbA1c in adults

				heterogeneity		
	NO	WMD (95% CI)	P within group	P heterogeneity	I^2^	P between subgroups
Subgroup analyses of chia on CRP						
Overall effect	6	–0.64 (–1.24, –0.04)	**0.03**	0.92	0%	
Trial duration (week)						
≥12	4	–0.81 (–1.50, –0.11)	**0.02**	0.91	0%	0.34
<12	2	–0.12 (–1.35, 1.10)	0.84	0.96	0%	
Intervention dose (g/day)						
>35	4	–0.81 (–1.50, –0.11)	**0.02**	0.9	0%	0.34
≤35	2	–0.12 (–1.35, 1.10)	0.84	0.96	0%	
Health status						
Overweight	4	–0.22 (–1.16, 0.71)	0.63	0.99	0%	0.25
T2DM	2	–0.94 (–1.73, –0.15)	**0.02**	0.90	0%	
Sex						
Both sexes	2	–0.94 (–1.73, –0.15)	**0.02**	0.90	0%	0.50
Male	1	–0.50 (–2.97, 1.97)	0.69	–	0%	
Female	3	–0.18 (–1.19, 0.83)	0.07	0.98	0%	
Subgroup analyses of chia on IL-6						
Overall effect	4	0.29 (–0.40, 0.98)	0.41	0.97	0%	
Trial duration (week)						
≥12	2	0.15 (–0.73, 1.04)	0.73	0.99	0%	0.63
<12	2	0.50 (–0.60, 1.59)	0.37	0.95	0%	
Intervention dose (g/day)						
>35	2	0.15 (–0.73, 1.04)	0.73	0.99	0%	0.63
≤35	2	0.50 (–0.60, 1.59)	0.37	0.95	0%	
Health status						
Overweight	4	0.29 (–0.40, 0.98)	0.41	0.97	0%	
T2DM	0	–	–	–	–	
Sex						
Both sexes	0	–	**–**	–	–	0.84
Male	1	0.16 (–1.27, 1.59)	0.82	–	00%	
Female	3	0.33 (–0.46, 1.11)	0.41	0.91	0%	
Subgroup analyses of chia on TNF-α						
Overall effect	4	0.05 (–0.21, 0.30)	0.72	0.70	0%	
Trial duration (week)						
≥12	2	0.10 (–0.17, 0.38)	0.45	0.95	0%	0.23
<12	2	–0.37 (–1.11, 0.37)	0.32	0.89	0%	
Intervention dose (g/day)						
>35	2	0.10 (–0.17, 0.38)	0.45	0.95	0%	0.23
≤35	2	–0.37 (–1.11, 0.37)	0.32	0.89	0%	
Health status						
Overweight	4	0.05 (–0.21, 0.30)	0.72	0.70	0%	
T2DM	0	–	–	–	–	
Sex						
Both sexes	0	–	–	–	–	0.85
Male	1	0.09 (–0.45, 0.63)	0.74	–	100%	
Female	3	0.03 (–0.26, 0.33)	0.82	0.50	0%	

Abbreviations: CI, confidence interval; WMD, weighted mean differences; T2D, type 2 diabetes; TC, total cholesterol.

### Effect of chia on IL-6

Pooling effect sizes from two publications (with 4 effect sizes), including 132 participants (69 cases and 63 controls), indicated that chia had no significant effect on IL-6, compared with placebo (WMD: 0.29 pg/dl; 95% CI: –0.40 to 0.98; P = 0.41), with no between-study heterogeneity (I^2^ = 0%, P = 0.90) (Fig. 2b).

### Effect of chia on TNF-α

Two publications (4 effect size), including 132 participants (69 cases and 63 controls), examined the effects of chia on TNF-α. The overall effect size showed that chia had no significant effect on TNF-α (WMD: 0.05 %; 95% CI: –0.21 to 0.30; P = 0.72), with no between-study heterogeneity (I^2^ = 0%, P = 0.70) (Fig. 2c). Subgroup analysis confirmed non-significance in all subgroups (Table 3).

### Sensitivity analysis

To ascertain each study’s impact on the overall effect size, we omitted each trial from the analysis, step by step. After deleting the study of Vuksan *et al.* 2007^(22)^ and Vuksan *et al.* 2017^(21)^ the overall effect of chia on CRP changed to (WMD: –0.49, CI 95%: –1.24, 0.25) and (WMD: –0.53, CI 95%: –1.22, 0.14). No significant effect was observed for IL-6 and TNF-α following sensitivity analysis.

### Quality assessment

The methodological quality and risk of bias for the eligible trials are detailed in Table 1. The majority of trials showed some concerns regarding their quality according to the RoB2 tool criteria. Specifically, four studies^(20,23)^ displayed a risk of bias categorized as ‘some concerns’, one study^(21)^ indicated a low risk of bias, and one study was identified as having a high risk of bias.^(22)^ The GRADE protocol was used to assess the certainty of the evidence (Table 4). The effect evaluates of CRP and TNF-α were regarded as moderate quality. The evidence for IL-6 was downgraded to low quality for serious heterogeneity and imprecision. The overall quality of the body of evidence of the present systematic review and meta-analysis was regarded as moderate.

**Table 4.** GRADE profile of chia on CRP, IL-6, and TNF-α in adults

Quality assessment						Summary of findings		Quality of evidence
Outcomes	Risk of bias	Inconsistency	Indirectness	Imprecision	Publication Bias	Number of intervention/control	WMD (95%CI)	
CRP	No serious limitations	serious limitations^a^	No serious limitations	No serious limitations	No serious limitations	106/104	–0.64 (–1.24, –0.04)	⊕⊕⊕irc; Moderate
IL-6	No serious limitations	serious limitations^b^	No serious limitations	serious limitations^c^	No serious limitations	69/63	0.29 (–0.40, 0.98)	⊕⊕irc;irc; Low
TNF-α	No serious limitations	No serious limitations	No serious limitations	serious limitations^c^	No serious limitations	69/63	0.05 (–0.21, 0.30)	⊕⊕⊕irc; Moderate

a The test for heterogeneity is significant, and the I^2^ is moderate, 53.3%.

b The test for heterogeneity is significant, and the I^2^ is moderate, 56%.

c Values are distributed within opposite direction across studies, CRP; C-Reactive Protein, IL-6; Interleukin-6, TNF-α; Tumour Necrosis Factor-alpha.

## Discussion

This meta-analysis aimed to examine the influence of chia consumption on three inflammatory markers: CRP, IL-6, and TNF-α in individuals with type 2 diabetes mellitus or overweight. The analysis involved four studies with 236 participants for CRP, two studies with 132 participants for IL-6, and two studies with 132 participants for TNF-α. The analysis of the effects of chia supplementation on CRP in patients with type 2 diabetes mellitus and overweight individuals revealed significant CRP reduction, particularly in those with a 12-week or longer intervention and an intervention dosage of over 35 grams per day. This suggests that the full effects of chia products might require a longer duration and higher doses to become apparent. The analysis did not reveal a significant effect of chia on IL-6 and TNF-α. Subgroup analysis also confirmed the lack of significance in all subgroups for TNF-α. This suggests that chia supplementation may not have a significant impact on these specific inflammatory markers in the studied populations. These results of the meta-analysis are consistent with some earlier studies that have suggested the consumption of chia may potentially reduce inflammation. For instance, a study by Vuksan *et al.* ^(24)^ reported that Salba-chia intervention reduced inflammatory factors like hs-CRP levels in overweight and obese adults with type 2 diabetes. Furthermore, in another study by Vuksan,^(25)^ 12-week dietary supplementation with the novel whole grain Salba (*Salvia hispanica L.*) was associated with decreased hs-CRP level and TNF-α but not IL6 in individuals with T2DM. While Nieman *et al.*,^(26,27)^ indicated that inflammation did not differ between chia seed (whole or milled) and placebo groups in overweight adults. Furthermore, Nikpayam *et al.* ^(14)^ revealed that supplementation of chia did not significantly affect hs-CRP and TNF-α. However, the significant variation seen among studies, possibly due to differences in study populations, methodologies, and treatment protocols, could explain some of the inconsistencies. In addition, Teoh *et al.* indicated that participants who consumed chia seed showed no significant difference in any of the inflammation markers compared to the control group.^(13)^ However, the study population was more diverse than our study, which could potentially undermine the results. The varying outcomes imply that the impact of consuming chia seeds may be influenced by contextual factors and may differ depending on an individual’s health condition and other related factors. Moreover, investigating the possible mechanisms that underlie the impact of chia seed consumption on various health outcomes could be valuable. For instance, chia seeds contain high levels of dietary fibre and omega-3 fatty acids, which could play a role in their potential health advantages. Conducting additional studies on the biological pathways by which chia seeds may influence inflammation, weight loss, and disease risk factors could provide insights into their potential health benefits and offer guidance for future interventions.

Moreover, a sensitivity analysis was carried out to determine the influence of each study on the overall effect size, and the results highlight the effect of removing two studies conducted by Vuksan *et al.* in 2007^(25)^ and 2017^(24)^ on the overall effect size of chia on CRP. So, it is recommended to approach the results of this meta-analysis with prudence, and further research is necessary to validate these outcomes.

There are mechanisms underlying the effects of chia on inflammation markers. Chia seeds contain high amounts of omega-3 fatty acids, specifically alpha-linolenic acid (ALA), which serves as a precursor to eicosapentaenoic acid (EPA) and docosahexaenoic acid (DHA), the two most extensively researched and recognized omega-3 fatty acids.^(28)^ Apart from omega-3 fatty acids and fibre, chia seeds also comprise polyphenols, which are plant-based substances possessing antioxidant and anti-inflammatory characteristics. Studies have demonstrated that polyphenols present in chia seeds, including caffeic acid and chlorogenic acid, possess anti-inflammatory properties by decreasing the production of pro-inflammatory cytokines such as TNF and IL-6.^(29)^ Polyphenols possess the capability to scavenge free radicals, which are molecules that can inflict harm on cells and lead to inflammation and chronic illnesses.^(30)^ Their capacity to improve blood sugar control and insulin sensitivity, both of which are linked to reduced levels of inflammation in the body, is one-way chia seeds might impact inflammation markers such as TNF, CRP, and IL-6.^(31)^

One explanation for the significant decrease in CRP but not in IL-6 and TNF-α could be the different biological pathways and sensitivities of these markers to dietary interventions. CRP is an acute-phase protein that responds rapidly to inflammation and may be more sensitive to dietary changes, whereas IL-6 and TNF-α are cytokines involved in chronic inflammation and might require more specific or potent interventions to show changes.^(32,33)^

This meta-analysis provides a comprehensive overview of the potential anti-inflammatory effects of chia seeds, integrating data from multiple randomized controlled trials. The study’s systematic approach and sensitivity analysis enhance the reliability of the findings, despite the limited number of included studies.

According to the assessment using the RoB2 tool criteria, the majority of the trials showed some concerns regarding their quality. This suggests that certain aspects of these studies raised potential biases or methodological limitations. The results revealed that four studies exhibited some concerns regarding bias, implying potential limitations or biases that could influence the reliability of their findings. On the other hand, one study demonstrated a low risk of bias, suggesting a relatively higher methodological quality. In contrast, one study was found to have a high risk of bias, indicating significant methodological limitations or biases that may impact the accuracy of its results. These findings highlight the importance of considering potential biases and limitations when interpreting the overall results of studies investigating the effects of chia on inflammatory markers. It is crucial for future research to address these methodological concerns to enhance the quality and validity of evidence in this field.

Our study was limited by the number of eligible studies, which may limit the generalizability of the findings. Also, the majority of the trials showed some concerns regarding their quality. Furthermore, the included studies varied in terms of sample size, intervention duration, and population characteristics, which may limit the comparability of the results. However, the certainty of the evidence was classified as low to moderate, which means that the true effect of chia on inflammatory markers is uncertain.

## Conclusion

This meta-analysis suggests that chia seed consumption may have a positive impact on reducing CRP levels, but does not show significant effects on IL-6 and TNF-α levels. Due to the limited number of studies and the overall low quality of evidence, several key areas for future research are highlighted. It is essential to conduct studies with larger sample sizes to enhance statistical power and reliability. Additionally, addressing the methodological limitations and potential biases identified through the RoB2 tool is crucial for improving evidence quality. Future research should also focus on determining the optimal dosage and duration of chia seed consumption to better understand its impact on inflammation. While chia seeds may offer some anti-inflammatory benefits, the current evidence is insufficient for definitive conclusions. Further investigation is needed to clarify the efficacy of chia seeds and elucidate the mechanisms behind their effects on inflammation.

## Supporting information

Pam et al. supplementary material Pam et al. supplementary material

## Data Availability

The data used to support the findings of this study are available from the corresponding author upon request.

## Supplementary material

The supplementary material for this article can be found at https://doi.org/10.1017/jns.2024.70
