# Supplementary material for: Chia seed supplementation and inflammatory biomarkers: a systematic review and meta-analysis
Source: J Nutr Sci. 2024 Dec 11;13:e91. doi: 10.1017/jns.2024.70 (PMC11658942; doi:10.1017/jns.2024.70)
Supplement: Pam et al. supplementary material [file S2048679024000703sup001.docx]

**supplementary table 1**

|  |  |  |
| --- | --- | --- |
| PubMed | "chia seed"[Title/Abstract] OR "chia seed extract"[Title/Abstract] OR "Salvia hispanica seed"[Title/Abstract] OR "Salvia hispanica"[Title/Abstract] | 394 |
|  | Intervention[Title/Abstract] OR "Intervention Study"[Title/Abstract] OR "Intervention Studies"[Title/Abstract] OR "controlled trial"[Title/Abstract] OR randomized[Title/Abstract] OR random[Title/Abstract] OR randomly[Title/Abstract] OR placebo[Title/Abstract] OR "clinical trial"[Title/Abstract] OR Trial[Title/Abstract] OR "randomized controlled trial"[Title/Abstract] OR "randomized clinical trial"[Title/Abstract] OR RCT[Title/Abstract] OR blinded[Title/Abstract] OR "double blind"[Title/Abstract] OR "double blinded"[Title/Abstract] OR trial[Title/Abstract] OR "clinical trial"[Title/Abstract] OR trials[Title/Abstract] OR "Pragmatic Clinical Trial"[Title/Abstract] OR "Cross-Over Studies"[Title/Abstract] OR "Cross-Over Study"[Title/Abstract] OR "Cross-Over"[Title/Abstract] OR parallel[Title/Abstract] OR "parallel study"[Title/Abstract] OR "parallel trial"[Title/Abstract] | 3,010,975 |
|  | #1 AND #2 | 53 |
| Scopus | TITLE-ABS-KEY ( "chia seed" OR "chia seed extract" OR "Salvia hispanica seed" OR "Salvia hispanica" ) | 1,218 |
|  | TITLE-ABS-KEY ( intervention OR "Intervention Study" OR "Intervention Studies" OR "controlled trial" OR randomized OR random OR randomly OR placebo OR "clinical trial" OR trial OR "randomized controlled trial" OR "randomized clinical trial" OR rct OR blinded OR "double blind" OR "double blinded" OR trial OR "clinical trial" OR trials OR "Pragmatic Clinical Trial" OR "Cross-Over Studies" OR "Cross-Over Study" OR "Cross-Over" OR parallel OR "parallel study" OR "parallel trial" ) | 7,331.117 |
|  | #1 AND #2 | 139 |
| Web of Science | "chia seed" OR "chia seed extract" OR "Salvia hispanica seed" OR "Salvia hispanica" (Topic) | 1,009 |
|  | "Intervention Study" OR "Intervention Studies" OR "controlled trial" OR randomized OR random OR randomly OR placebo OR "clinical trial" OR Trial OR "randomized controlled trial" OR "randomized clinical trial" OR RCT OR blinded OR "double blind" OR "double blinded" OR trial OR "clinical trial" OR trials OR "Pragmatic Clinical Trial" OR "Cross-Over Studies" OR "Cross-Over Study" OR "Cross-Over" OR parallel OR "parallel study" OR "parallel trial" (Topic) | 4,808,743 |
|  | #1 AND #2 | 103 |
| Cochrane | "chia seed" OR "chia seed extract" OR "Salvia hispanica seed" OR "Salvia hispanica" in Title Abstract Keyword | 51 |
|  | "Intervention Study" OR "Intervention Studies" OR "controlled trial" OR randomized OR random OR randomly OR placebo OR "clinical trial" OR Trial OR "randomized controlled trial" OR "randomized clinical trial" OR RCT OR blinded OR "double blind" OR "double blinded" OR trial OR "clinical trial" OR trials OR "Pragmatic Clinical Trial" OR "Cross-Over Studies" OR "Cross-Over Study" OR "Cross-Over" OR parallel OR "parallel study" OR "parallel trial" in Title Abstract Keyword | 8984 |
|  | #1 AND #2 | 46 |
